# Random fields and apparent exchange bias in the dilute Ising antiferromagnet Fe_0.6_Zn_0.4_F_2_

**DOI:** 10.1038/s41598-020-71533-6

**Published:** 2020-09-03

**Authors:** D. C. Joshi, P. Nordblad, R. Mathieu

**Affiliations:** Department of Materials Science and Engineering, Uppsala University, Box 35, 751 03 Uppsala, Sweden

**Keywords:** Condensed-matter physics, Magnetic properties and materials

## Abstract

Random field induced spontaneous excess moments appear in field cooled single crystals of diluted Ising antiferromagnets. Here we report results from low temperature measurements of field cooled (including zero field) magnetic hysteresis loops parallel and perpendicular to the *c*-axis of a single crystal of composition Fe_0.6_Zn_0.4_F_2_. We find that weak static ferromagnetic excess moments attained on field cooling give rise to an apparent exchange bias of the magnetic hysteresis loops, whose magnitude is controlled by temperature and the strength and direction of the cooling field. Random field induced temporal excess moments only become observable in cooling fields larger than 1 T applied along the *c*-axis direction of the Fe_0.6_Zn_0.4_F_2_ single crystal.

## Introduction

The antiferromagnet FeF_2_^1^ is a physical realization of a model 3d Ising system^2^ as well as an important spintronic material^3,4^. At temperatures below the Néel temperature, T_N_ = 78.4 K, of FeF_2_ an excess magnetic moment develops, that near T_N_ decays with a critical exponent characteristic of 3d Ising systems^5^. This excess moment gives rise to an apparent exchange bias associated with the vertical shift of the hysteresis loops occurring when cooling the sample through T_N_ in a finite magnetic field^6^. The excess moment is rigidly locked to the cooling field direction and is virtually unaffected by any magnetic field changes in the antiferromagnetic state. This is reflected in a field dependent rapidly saturating thermo-remnant magnetization (TRM) and zero isothermal remnant magnetization (IRM) at all accessible fields and temperatures below T_N_^6^. A similar behavior of the TRM and IRM has been observed in antiferromagnetic Co_3_O_4_ nanowires^7,8^, results that are discussed and compared to corresponding results on the dilute antiferromagnet Fe_1-x_Zn_x_F_2_^9^.

A dilute Ising antiferromagnet in a homogeneous magnetic field (DAFF)^10–12^ is a replica of the random field Ising model (RFIM)^13^. FeF_2_ diluted by diamagnetic dopant Zn (Fe_1-*x*_Zn_*x*_F_2_ (FZF)) has been extensively used as experimental model systems of a random exchange Ising model (REIM) system in zero applied field and RFIM system in an applied field^14–18^. Measurements of the field and time dependence of the remnant magnetization of FZF^18–21^ have revealed characteristics of RFIM systems superposed on an additional low field static remnant moment comparable to that of FeF_2_.

In this article, we examine the temperature and field dependence of the parallel and perpendicular magnetization of a Fe_0.6_Zn_0.4_F_2_ single crystal with special emphasis on apparent exchange bias and the effects of random fields by comparing the parallel and the perpendicular magnetization behaviors.

## Results and discussion

**Figure 1:**
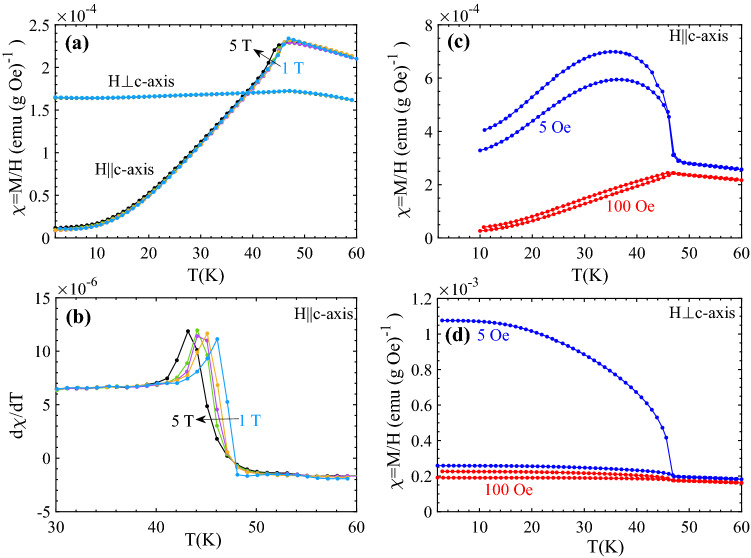
(**a**) Temperature dependence of magnetic susceptibility χ = M/H recorded after ZFC, and after FC in higher magnetic fields (1 T ≤ H ≤ 5 T) for magnetic fields parallel (||*c*) and perpendicular (⊥*c*) to the *c*-axis of the Fe_0.6_Zn_0.4_F_2_ single crystal. (**b**) The temperature gradient of susceptibility (dχ/dT) of ZFC data for H||*c*-axis plotted as a function of temperature. (**c**) and (**d**) M(T)/H after ZFC (lower curves) and FC (upper curves) the sample to 2 K recorded in H = 5 Oe (blue circles) and H = 100 Oe (red circles) applied parallel (**c**) and perpendicular (**d**) to the *c*-axis of the Fe_0.6_Zn_0.4_F_2_ single crystal.

Figure 1a shows the temperature dependence of the ZFC parallel and perpendicular susceptibility at different high magnetic fields. The Néel temperature is found at the temperature where d(M(T)/H)/dT has a maximum, see Fig. 1b. The observed low field T_N_ ~ 48 K and the decrease of T_N_ with increasing field accords with earlier findings on samples of the same composition^22^. The H dependence of T_N_ stems from the random fields effect induced in DAFF^23^. It is interesting to note that the differential susceptibility (Δχ = χ_||_ − χ_⊥_) has somewhat larger relative amplitude than that of pure FeF_2_ (see Fig. 7 in Ref.^1^) and that the Néel temperature decreases linearly from that of FeF_2_ (T_N_ = 78.4 K) with increasing *x* as has been observed earlier^24,25^.

Figure 1c,d shows the temperature dependence of the low field magnetic susceptibility χ = M/H recorded after ZFC and FC protocol in H = 5 Oe and 100 Oe for two different orientations, parallel (||*c*) (Fig. 1c) and perpendicular (⊥*c*) to the *c*-axis (Fig. 1d) for Fe_0.6_Zn_0.4_F_2_ single crystal. The overall susceptibility χ(T) behavior at low fields is quite similar to that of the FeF_2_ single crystal reported in Ref.^6^. The irreversibility between the ZFC and FC curves below T_N_ provides the signature of the excess moments associated with uncompensated antiferromagnetic sub-lattices. In the case of FeF_2_, the excess moment supposedly arises from a distortion of the antiferromagnetic domain structure of piezo magnetic origin, which also has been suggested as the origin of the low field frozen in excess moments of FZF^19^.

**Figure 2:**
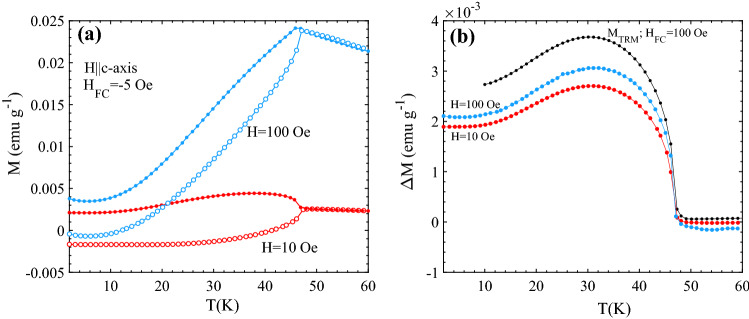
(**a**) Temperature dependence of the magnetization M after field cooling in H_FC_ = − 5 Oe (M_-5Oe_) and recorded in H = 10 Oe and 100 Oe during heating (open circles) and cooling (closed circles) for H||*c*-axis. (**b**) Difference between M(T) recorded in heating (H_FC_ = − 5 Oe) and cooling in H = 10 and 100 Oe; ΔM = M_FC_(T) − M_-5Oe_(T))/2 plotted together with M_TRM_(T) (cooling field 100 Oe) for H||*c*-axis.

Even though we have used the ultra-low field option before performing all these measurements, a small stray/remnant field was still present in our experiment. Because of this, the observed ZFC (cooled in a stray magnetic field) curves contain a finite excess moment directed along the stray field (see also Fig. SM7 in supplementary material of Ref.^6^). This effect is demonstrated in Fig. 2a, where the temperature dependence of M(T) was recorded after cooling the sample in a low negative magnetic field H_FC_ = − 5 Oe and measured under fields H = 10 Oe and 100 Oe during the heating and cooling cycle for H||*c*-axis. When cooled in – 5 Oe, the excess moment (M_exc_) is almost saturated and directed in the negative magnetization direction. Applying a positive field does not change the direction of M_exc_. Thus, in the M(T) experiments on increasing temperature, the measured M_FC_(T) = χ_||_H + M_exc_(T) in the FC case and M_-5Oe_(T) = χ_||_(T)H − M_exc_(T) when cooled in − 5 Oe. The difference between the cooling and heating curve at a particular temperature is then equal to 2 × M_exc_(T). This is demonstrated in Fig. 2b, where (M_FC_(T) − M_-5Oe_(T))/2 is plotted together with the measured M_TRM_(T) after FC in 100 Oe. The somewhat smaller magnitude of the derived M_exc_(T) compared to M_TRM_(T) is due to the fact that the excess moment is only almost saturated when field cooling in − 5 Oe.

**Figure 3:**
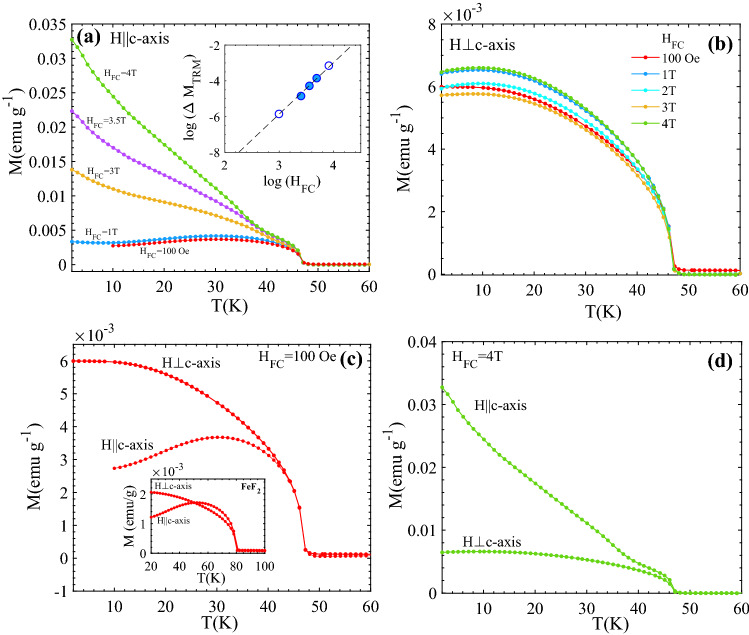
Temperature dependence of thermo-remnant magnetization (TRM) measured along (**a**) parallel (||*c*) and (**b**) perpendicular (⊥*c*) to the *c*-axis of the Fe_0.6_Zn_0.4_F_2_ single crystal. The spurious susceptibility contribution to the magnetization due to a weak residual magnetic field in the superconducting magnet has been subtracted from curves. The inset of (**a**) shows a log–log plot of ΔM_TRM_(10 K) (= M_TRM_(H_FC_,10 K) − M_TRM_(1 T, 10 K)) vs. H_FC_ for H||*c*-axis. The solid symbols represent the data points obtained from the main figure (H_FC_ = 4 T, 3.5 T and 3 T), while the hollow symbols are collected from supplementary Fig. SM4. The dashed line (slope 2.8) is a guide to the eye of the predicted field dependence. (**c**) and (**d**) show M_TRM_(T) parallel and perpendicular to the *c*-axis measured after field cooling in H_FC_ = 10 mT (100 Oe) and 4 T, respectively. The inset of (**c**) shows the corresponding curves for a FeF_2_ single crystal (H_FC_ = 100 Oe (the H_FC_ = 4 T curves are in both directions almost identical to the H_FC_ = 100 Oe curves for FeF_2_).

The signatures of random fields are observed in the temperature dependence of thermo-remnant magnetization M_TRM_(T). The M_TRM_(T) curve for H (≥ 1 T) applied along the *c*-axis (H||*c*-axis) shows a continuous non-linear increase at low temperatures (T < T_N_) due to cumulative effect of low field static excess moments and random field induced remnant moments (Fig. 3a). Whereas, for low fields (H = 100 Oe) and for all fields in the case of H⊥*c*-axis only the saturated static excess moment is observed and M_TRM_(T) curves (Fig. 3a–c) are quite similar to the corresponding curves of FeF_2_ (inset of 3c)^6^. See supplementary Fig. SM2 for M_TRM_(T) curves represented in µ_B_/Fe for two selected H_FC_ = 10 mT and 4 T. A comparison of M_TRM_ curves for both orientations under H_FC_ = 4 T are shown in Fig. 3d. The logarithmic dependence of ΔM_TRM_(= M_TRM_(H_FC_, 10 K) − M_TRM_(H_FC_ = 1 T, 10 K)) on log(H_FC_) for H||*c*-axis (shown in the inset of Fig. 3a) follows a linear behavior with a slope of ~ 2.8, which is in agreement with the predicted behavior due to random field effects and in agreement with earlier findings^18^.

**Figure 4:**
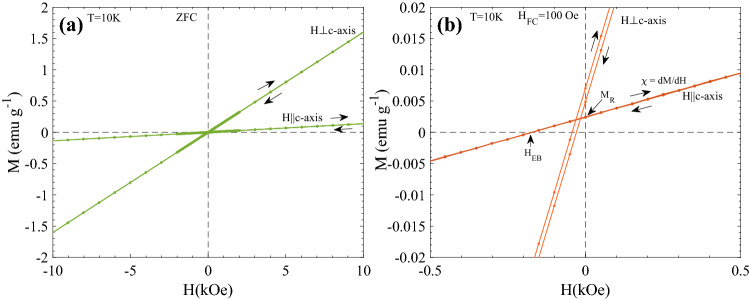
(a) the field dependence of magnetization M(H) curves recorded with (**a**) field sweep 0 →  + 1 T → − 1 T →  + 1 T after ZFC from 60 K down to 10 K, for two different orientations. (**b**) M(H) recorded with field sweep 100 Oe →  + 1 T → − 1 T →  + 1 T after FC in 100 Oe from 60 K down to 10 K. The parameters H_EB_, M_R_, and χ are indicated in (**b**). The weak (~ 5 Oe) reversed coercivity observable for the H⊥*c* loop is an artefact due to the field history dependent residual field in the superconducting magnet of the MPMS SQUID magnetometer—the same coercivity artefact is seen also in the zoomed M vs H curves in Fig. 5.

**Figure 5:**
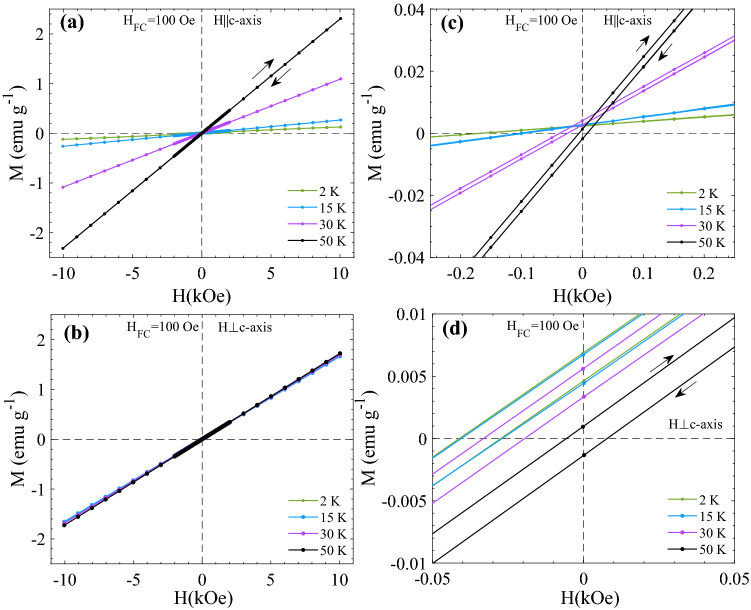
M(H) curves recorded with field sweep 100 Oe →  + 1 T → − 1 T →  + 1 T after FC in 100 Oe from 60 K down to different temperatures, for (**a**) H||*c*-axis and (**b**) H⊥*c*-axis. (**c**) and (**d**) are zoomed views of (**a**) and (**b**), respectively.

**Figure 6:**
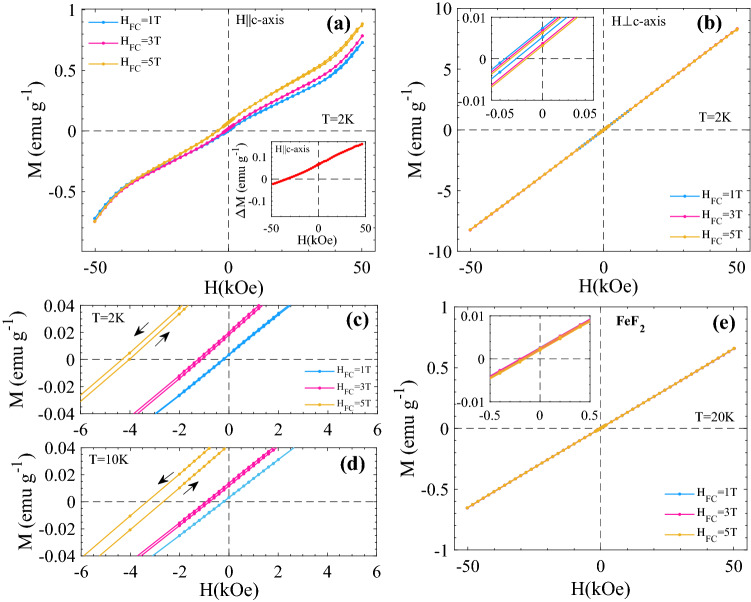
M(H) recorded after FC with field sweep + H_FC_ → 5 T → − 5 T → 5 T from 60 K down to T = 2 K for (**a**) H||*c*-axis and (**b**) H⊥*c*-axis. Inset of (**a**) shows the H dependence of difference between the M(H) curves measured after field cooling in H_FC_ = 5 T and after H_FC_ = 1 T for H||*c*-axis. Inset of (**b**) shows the zoomed view of main panel loops. (**c**) and (**d**) shows the zoomed view of M(H) loops with field sweep + H_FC_ → 5 T → − 5 T → 5 T after field cooling from T = 60 K to 2 K and 10 K, respectively (see supplementary Fig. SM4 for full M(H) curves). (**e**) M(H) loops with field sweep + H_FC_ → 5 T → − 5 T → 5 T measured ||*c*-axis after field cooling from T = 100 K to T = 20 K for an FeF_2_ single crystal.

Figure 4a shows the M(H) curves recorded at T = 10 K with field sweep 0 → + 1 T → − 1 T → + 1 T after ZFC for H||*c*-axis and H⊥*c*-axis. A shift in the hysteresis loops (apparent exchange bias^6^) is observed for both orientations when the sample is cooled from T = 60 K to T = 10 K in H_FC_ = 100 Oe as shown in Fig. 4b. The observed shift |H_EB_| is 36 Oe and 170 Oe for ⊥ c and || c orientations, respectively. The effect of temperature on the FC M(H) curves are shown in Fig. 5. The M(H) curves are recorded after FC in H_FC_ = 100 Oe from 60 K down to different temperatures with field sweep 100 Oe → + 1 T → − 1 T → + 1 T. Zoomed views of the M(H) curves at low fields are shown in Fig. 5c,d. A significant decrease in the slope χ = dM/dH and increase in the apparent exchange bias H_EB_ are observed as the measuring temperature decreases from T = 60 K → 2 K, for H||*c*-axis. However, for H⊥*c*-axis, these changes are very subtle as compared to H||*c*-axis. In Fig. 6 M(H) curves recorded after cooling the sample form 60 K to 2 K under different cooling fields with field sweep + H_FC_ → 5 T → − 5 T → 5 T are shown for (a) H||*c*-axis and (b) H⊥*c*-axis (see supplementary Fig. SM4 for the corresponding T = 10 K data). In contrast to H⊥*c*-axis, M(H) curves for H||*c*-axis show non-linear asymmetric behavior. This behavior becomes more prominent with increasing H_FC_ and decreasing T. This trend can be clearly noticed from the absolute value of M at maximum fields (± 5 T) for T = 2 K and 10 K (see inset of supplementary Fig. SM4b). The difference plot of the M(H) curves recorded after FC in H_FC_ = 5 T and H_FC_ = 1 T (∆M = M(H_FC_ = 5 T,H) − M(H_FC_ = 1 T,H)) shown in inset of Fig. 6a confirm the upward shift of the 5 T curve, compared to the 1 T curve, due to random field induced excess moments. Interestingly, it also implies an enhanced overall response to magnetic field changes of the sample when cooled in 5 T, compared to the M vs H curve of the sample when cooled in a lower field (1 T), where random field induced excess moments are hidden by the larger static excess moments. Another observation is that temperature dependent relaxation effects yield intrinsic coercivity changes with increasing H_FC_, as illustrated in Fig. 6c,d (note the direction of the arrows marking the magnetic field sweeps in those panels in comparison to the low-field results presented in Fig. 4). The observed temporal intrinsic coercivity H_C_ is 330 and 140 Oe at 10 K and 2 K, respectively, for H_FC_ = 5 T. Whereas, the small spurious coercivity noticed in ZFC and H_FC_ = 1 T M(H) curves is associated with the artifact due to the field history dependent residual field in the superconducting magnet of the SQUID magnetometer (see Fig. 4 and supplementary Fig. SM5). Neither non-linearities nor intrinsic coercivity enhancement is observed in undoped FeF_2_ in the same magnetic field range, as illustrated in Fig. 6e. The results of ZFC M vs. H measurements H||*c*-axis up to fields of 18 T on Fe_0.6_Zn_0.4_F_2_^22^ indicate that the hysteresis properties of a sample that has been field cooled in very high magnetic field (e.g. 18 T) should show interesting new features compared to the ZFC sample.

**Figure 7:**
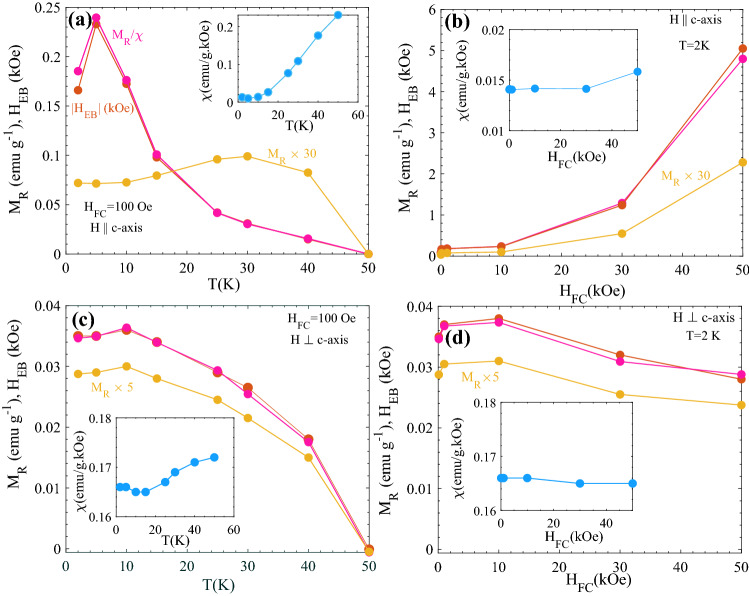
Variation of |H_EB_|, M_R_/χ and M_R_, determined from Fig. 5 and 6 for (**a**,**b**) H||*c*-axis and (**c**,**d**) H⊥*c*-axis as a function of the (**a**–**c**) temperature and (**b**–**d**) H_FC_. The insets show the corresponding variation of χ = dM/dH (in (**b**), where the M vs H loops are non-linear (Fig. 6a) at higher fields, χ is defined from (dM/dH)_H=0_).

**Figure 8:**
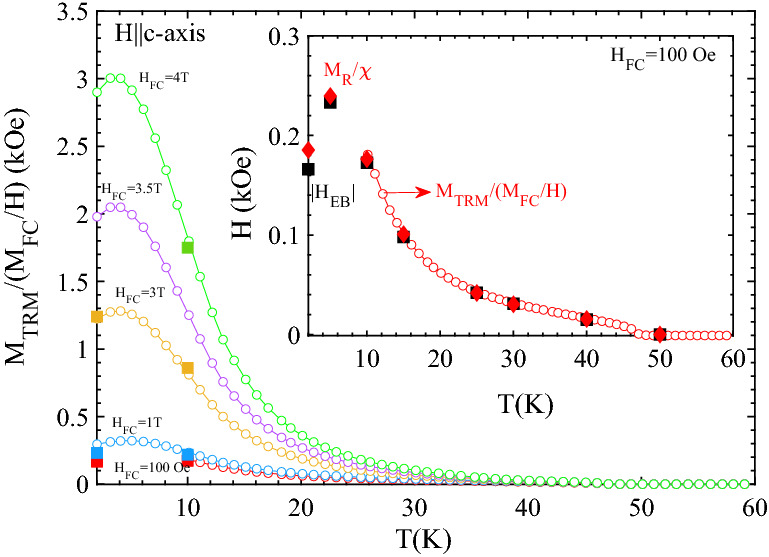
Temperature dependence of the ratio of M_TRM_(H_FC_) (from Fig. 3a) divided by the susceptibility M_FC_/H recorded at H = 1 T (Fig. 1a) for H||*c*-axis. For comparison |H_EB_| determined from the M(H) curves (Fig. 6a and supplementary Fig. SM4a) is shown by square symbols. Inset shows M_TRM_/(M_FC_/H) (open red circles), |H_EB_| (solid black squares) and M_R_/χ (solid red prisms) for H_FC_ = 100 Oe (cf. Fig. 7a).

The linear M vs H curves observed for fields H⊥*c*-axis and low fields H||*c*-axis can be described by the equation: M = χH + M_R_, yielding H_EB_ = − M_R_/χ at M = 0. For comparison to this simple description, the parameters H_EB_, M_R_ and χ(inset) extracted from Figs. 5 and 6 are plotted in Fig. 7 for both H||- and ⊥*c*-axis. The behavior of M_R_/χ is consistent with the observed values of the apparent H_EB_. For H||*c*-axis the M_R_ dependence at higher cooling fields accords with that predicted for RFIM systems^18^. As seen in Fig. 7b, H_EB_ value approaches ~ 5 kOe at 2 K (H_FC_ = 5 T). These observations are summarized in Fig. 8. Figure 8 shows the temperature dependence of the ratio of M_TRM_(H_FC_) (from Fig. 3a) divided by the susceptibility M_FC_/H recorded at H = 1 T (Fig. 1a) for H||*c*-axis. For comparison |H_EB_| determined from the M(H) curves (Fig. 6a and supplementary Fig. SM4a) is shown by square symbols. The inset shows a comparison of M_TRM_/(M_FC_/H), |H_EB_| and M_R_/χ for H_FC_ = 100 Oe.

## Conclusions

The field and temperature dependence of the magnetization measured parallel and perpendicular to the *c*-axis of the dilute Ising antiferromagnet Fe_0.6_Zn_0.4_F_2_ reveal apparent exchange bias effects governed by static excess moments, which at higher fields in the case of H||*c*-axis are enhanced by random field induced temporal excess moments. H_EB_ amounts to ~ 5kOe at 2 K (H_FC_ = 5 T). The observed hysteresis behavior with field H⊥*c*-axis and at low fields H||*c*-axis is similar to that observed in FeF_2_^6^, indicating that the origin of static excess moments is the same in pure and diluted samples. Irrespective of origin of the excess moments, the vertical shift (apparent exchange bias) of the hysteresis curves is directly reflected in the field dependence of the TRM(H) and IRM(H) curves^7–9^; where IRM(H) = 0 at all accessible field, i.e. no remnant magnetization is attained on a zero field cooled sample. Systems with such properties exhibit magnetic hysteresis curves with apparent exchange bias that are fully controlled by the cooling field and the measurement temperature. The temporal excess moment give rise to cooling field and field sweep rate dependent intrinsic coercivity that of the order of ~ 330 Oe at 10 K.

## Methods

The temperature and field dependent magnetization measurements for Fe_0.6_Zn_0.4_F_2_ single crystal were performed using a superconducting quantum interference device (SQUID) magnetometer from Quantum Design Inc. (Model: XL). The same single crystal was used in Ref.^5^. The magnetic field H was applied along two different orientations of the single crystal (*i*) parallel to the *c*-axis (H||*c*-axis) and (*ii*) perpendicular to the *c*-axis (H⊥*c*-axis). The temperature dependence of the magnetization M(T) was recorded in zero field cooled (ZFC) and field cooled (FC) conditions in different magnetic fields. The thermo-remnant magnetization (TRM) was recorded on warming in zero magnetic field, after cooling the sample from 60 K down to 2 K in presence of an applied magnetic field. The schematic of M(T) measurement is shown in supplementary Fig. SM1. The field dependence of the magnetization M(H) was recorded at a temperature T after zero-field cooling from 60 K down to low temperatures (ZFC M(H)) and after field cooling in H_FC_ (FC M(H)). For the FC M(H) measurements, the field is increased from H_FC_ to the maximum field (H_max_) then decreased to − H_max_ and finally increased back to H_max_. Before performing all of the ZFC or FC measurements with H_FC_ ≤ 100 Oe, the background magnetic field of the magnet was reset to zero by using the ultra-low field option.

## Supplementary information

Supplementary file1
