# Supplementary material for: Random fields and apparent exchange bias in the dilute Ising antiferromagnet Fe0.6Zn0.4F2
Source: Sci Rep. 2020 Sep 3;10:14588. doi: 10.1038/s41598-020-71533-6 (PMC7471909; doi:10.1038/s41598-020-71533-6)
Supplement: Supplementary file 1 — Supplementary file1 [file 41598_2020_71533_MOESM1_ESM.pdf]

Random fields and apparent exchange bias in the dilute Ising antiferromagnet $Fe_{0.6}Zn_{0.4}F_2$

D. C. Joshi[1*], P. Nordblad[1], R. Mathieu[1]

[1]*Department of Materials Science and Engineering, Uppsala University, Box 35, SE-751 03, Uppsala, Sweden*

*Corresponding author: deep.joshi@angstrom.uu.se

Supplementary Material

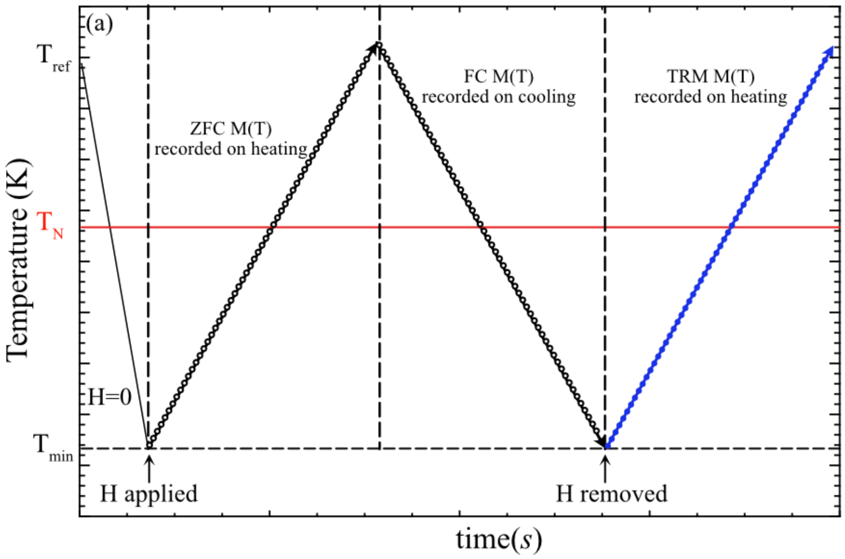

Fig. SM1: Schematic of the temperature dependent magnetization M(T) measurement experiment protocol, illustrating the cooling and reheating of a ZFC measurement, FC on cooling (black curve) and TRM (blue curve) recorded on heating after removing the magnetic field. Although these are shown sequently in the figure each of these measurement can perform independently, e.g. in the case of TRM, by rapidly cooling the sample in a magnetic field down to the lowest temperature and recording the magnetization in reheating in zero magnetic field.

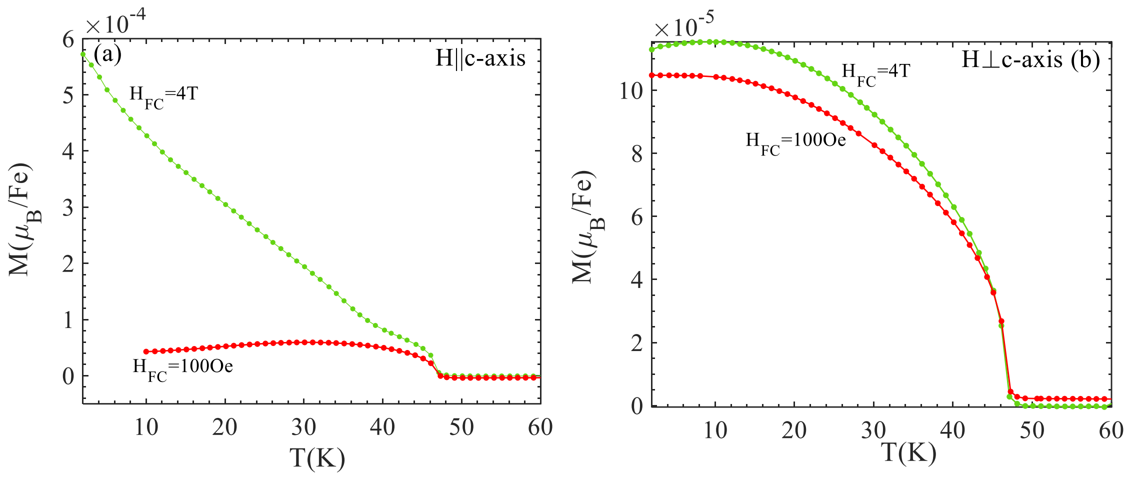

Fig. SM2: Temperature dependence of thermo-remnant magnetization (TRM) of Fig. 3 represented in μ_B/Fe for two selected cooling fields; H_{FC}=10 mT (100 Oe) and 4 T.

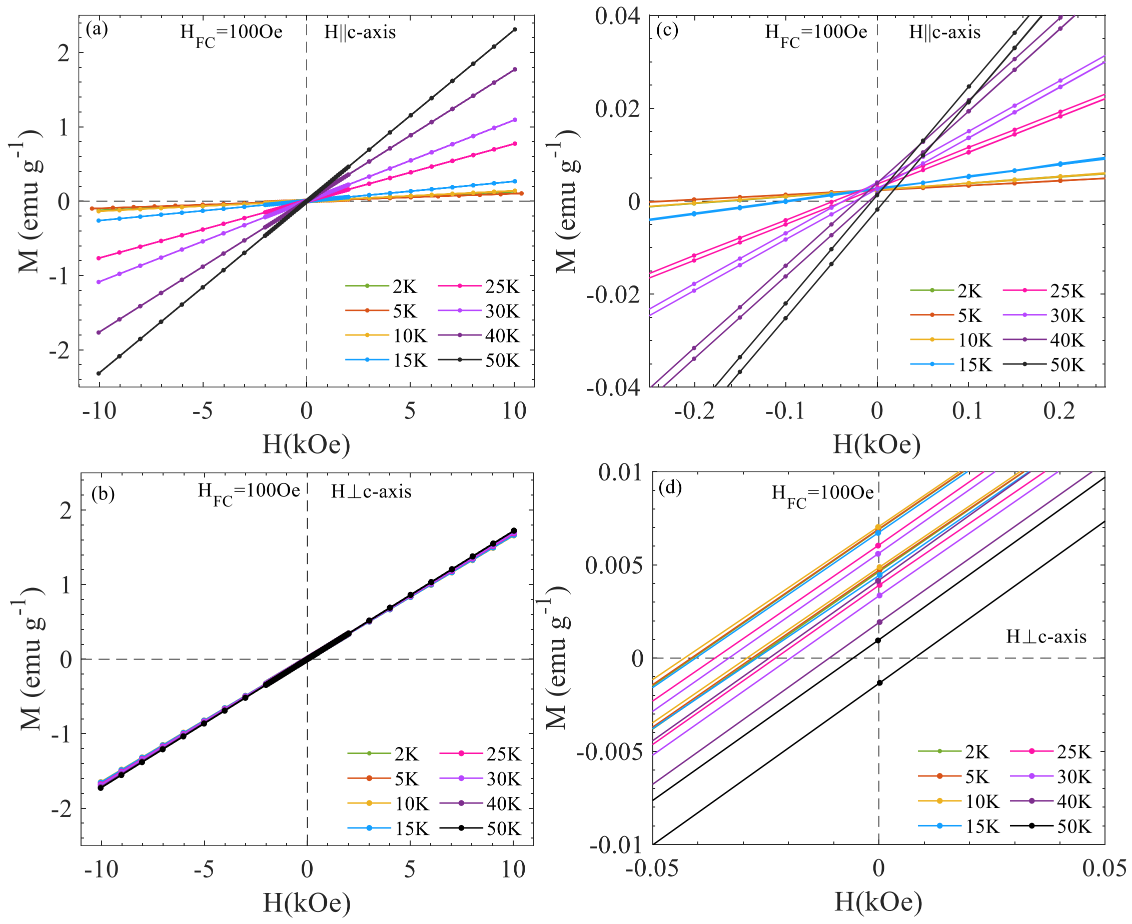

Fig. SM3: M vs. H loops similar to those of Fig. 5 in the article, but including all measured temperatures.

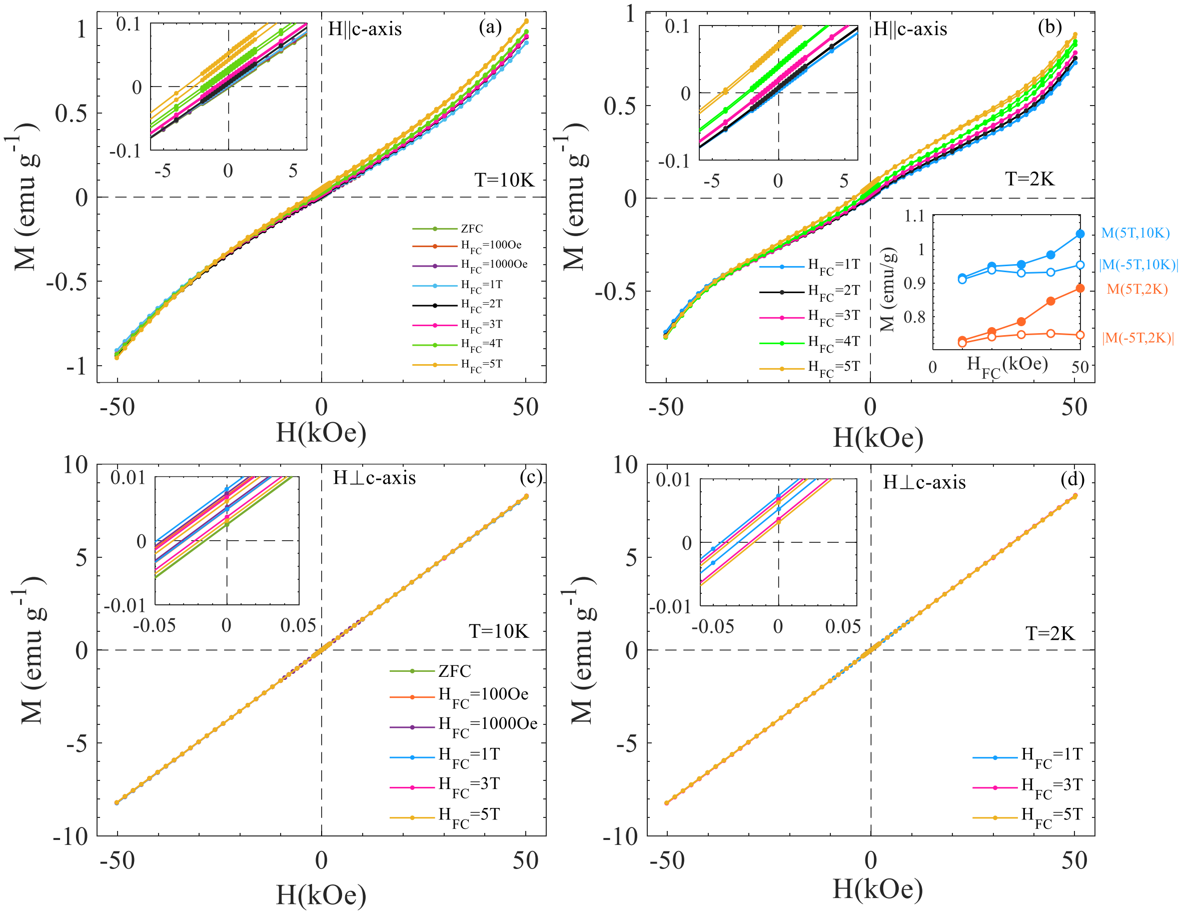

Fig. SM4: M vs. H measured after cooling in different fields (H_{FC}) at 10 K and 2 K. (a) and (b) parallel *c*-axis, upper insets zooms of the low field parts and lower inset in (b) magnetization values at +5 T and – 5 T vs. H_{FC}. (c) and (d) corresponding curves for fields perpendicular to the *c*-axis.

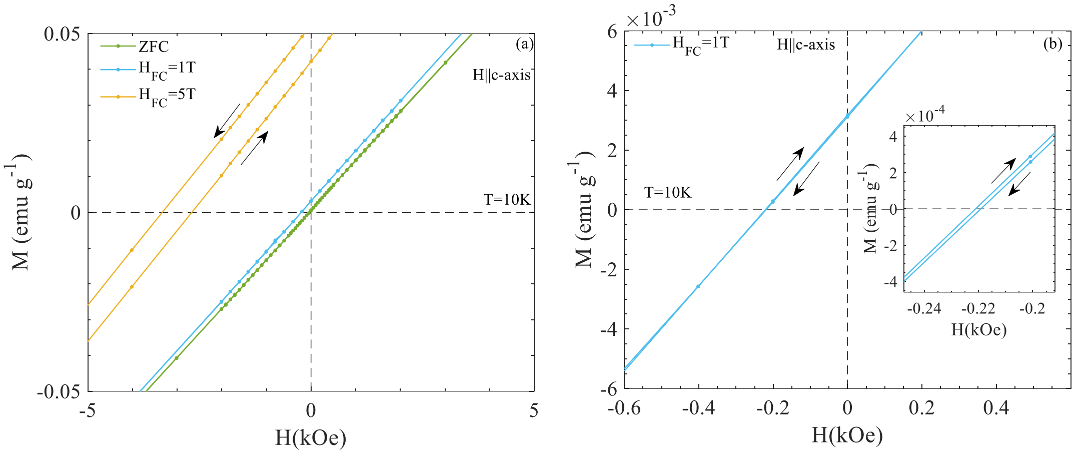

Fig. SM5: (a) M(H) loops with field sweep $+H_{FC} \rightarrow 5$ T$\rightarrow -5$ T$\rightarrow 5$ T after field cooling from T=60 K to 10 K for $H_{FC} = 1$ T and 5 T plotted together with ZFC M(H) loop. The data for $H_{FC} = 1$ T is shown in (b). Inset shows the zoomed view of the main panel.
